# Homeopathy or Allopathy

**Published:** 1897-04

**Authors:** 


### HOMEOPATHY OR ALLOPATHY.

The *New England Medical Monthly,* in answer to a statement in *Life's* columns, asks:

By the way, does *Life* really know of any surgeon who removes the appendix on general principles?

*Life* does.

Dr. ——, of New York, than whom no allopath in this city is oftener named in this connection, advocates the removal of the appendix from children when fifteen days old.

Dr. ——, one of the most swollen of swell old-school doctors in this city, was recently operating before students. He was at work in the abdominal cavity. Coming to the appendix, he said: "This is of no use to the patient; it may be of harm. While we are here we might as well remove it, and insure against future trouble." It was perfectly healthy. He removed it.

Our esteemed contemporary then quotes us—for our demolition, of course:

"The joke of it is that during all this reign of blood and terror the homeopaths, it appears, have been quietly treating it (appendicitis) medicinally, seldom operating and rarely losing a case."

Just how "rarely" is not stated, but we will supply the information by saying that the proportion of fatal cases under any sort of medical treatment has been shown to be something over twenty-five per cent. from causes which cannot be reached by medical treatment—concretions, tuberculosis, empyema, abscess, etc. The death rate of appendicitis under the best surgical treatment has been shown to be less than one per cent., or almost no death rate at all.

Lack of space prevents our indulgence in lengthy details, but we will mention one homeopath, who has practiced in this city twenty-five years, seeing his due share of appendicitis cases in his own practice, besides those brought to his notice by fellow-practitioners—he being a surgeon with college and hospital standing. He has never lost a case and never failed to cure a case, whether primary or recurrent, with strict homeopathic prescribing.

Other veterans tell the same story. Occasionally, at very long intervals, a patient is lost. The percentage of deaths among these homeopaths is not twenty-five per cent.—onequarter of one per cent. would be a liberal estimate. We regret that our esteemed contemporary should "give away" the allopath in this thoughtless manner, but that twenty-five per cent. must refer to practice other than homeopathic.

By the way, where shall we tabulate the cases mentioned every day in the daily press of eminent men who are operated upon, and die in a day or two? Probably not included in the "less than one per cent."

While not the advocate of any school, *Life* is still unable to resist the belief that those who are really attached to their appendix—in any sense—will find the safest and most comfortable traveling on the homeopathic highway. All the guide-posts point in that direction.—*Life*, March 4th, 1897.

